# Investigation of Oxide Layer Development of X6CrNiNb18-10 Stainless Steel Exposed to High-Temperature Water

**DOI:** 10.3390/ma17184500

**Published:** 2024-09-13

**Authors:** Georg Veile, Radhika Hirpara, Simon Lackmann, Stefan Weihe

**Affiliations:** 1 Materials Testing Institute, University of Stuttgart, Pfaffenwaldring 32, 70569 Stuttgart, Germany; 2 Max Planck Institute for Intelligent Systems, Heisenbergstraße 3, 70569 Stuttgart, Germany

**Keywords:** stainless steel, SEM, TEM, XPS, high temperature corrosion, passive films

## Abstract

The oxide layer development of X6CrNiNb18-10 (AISI 347) during exposure to high-temperature water has been investigated. Stainless steels are known to form a dual oxide layer in corrosive environments. The secondary Fe-rich oxide layer has no significant protective effect. In contrast, the primary Cr-rich oxide layer is known to reach a stabilized state, protecting the base metal from further oxidation. This study’s purpose was to determine the development of oxide layer dimensions over exposure time using SEM, TEM and EDX line scans. While a parabolic development of Cr in the protective primary layer and Fe in the secondary layer was observed, the dimensions of the Ni layer remained constant. Ni required the presence of a pronounced Fe-rich secondary layer before being able to reside on the outer secondary layer. With increasing immersion time, the Ni element fraction surpassed the Cr element fraction in the secondary layer. Oxide growth on the secondary layer could be observed. After 480 h, nearly the entire surface was covered by the outer oxide layer. In the metal matrix, no depletion of Cr or Ni could be observed over time; however, an increased presence of Cr and Ni in the primary layer was found at the expense of Fe content. The Nb-stabilized stainless steel was subject to the formation of Niobium pentoxide (Nb_2_O_5_), with the quantity and magnitude of element fraction increasing over exposure time.

## 1. Introduction

Reactor interiors and pipelines of Nuclear Power Plants (NPP) are exposed to transient loading. High-temperature water (HTW), used for cooling, reduces the fatigue limit due to corrosive effects. Stainless Steel (SS) is used for reactor interiors or pipes due to its corrosion resistance and weldability [1,2,3]. Type 300 SS, such as AISI 304 with a high chromium content (>17 wt.%), is particularly suitable due to the formation of a protective Cr-rich oxide layer on the surface exposed to HTW [4]. Ni is also added to the alloy to improve austenite stability [5,6]. On the other hand, these alloys are susceptible to intergranular stress corrosion cracking [7,8,9]. The chromium in austenitic SS has a high affinity to carbon, resulting in the formation of chromium carbide. The formation of chromium carbides at grain boundaries depletes the chromium in the austenitic matrix, thereby increasing the susceptibility to intergranular corrosion. This phenomenon is known as sensitization, and it drains the austenitic matrix while reducing corrosion resistance and being more susceptible to intercrystalline corrosion. Niobium, as an alloying element in X6CrNiNb18-10, helps to prevent sensitization by forming niobium carbides instead of chromium carbides, thus enhancing corrosion resistance [10].

Nb-stabilized nuclear grade X6CrNiNb18-10, also known as AISI 347, has seen application in German Boiling Water Reactors (BWR) due to its superior resistance to intergranular stress corrosion cracking [2,8,11]. The SS oxide layers are known to build a dual-layer structure in HTW, with a protective Cr-rich inner layer and an Fe-rich secondary layer [12]. Fulger et al. states that the outer, secondary oxide layer is porous, and thus less protective compared to the inner compact oxide layer [4]. According to reference [12], it was found that both layers contained pores and other defects. Furthermore, in [13], it was found that the secondary oxide layer peeled off during transient thermal loading and the associated deformation of pipes in HTW. With regard to fatigue tests in medium conditions, the existence of a well-formed primary oxide layer before the start of the test is a common precondition. The precondition prior to fatigue tests was described in the conference contributions of references [14,15], which formed the basis of this work. Prior to that, various studies [4,7,16,17,18,19] have dealt with the development of the oxide layer morphology and the oxide layer thickness of SS in HTW. Fulger et al. investigated AISI 304L, where they found that the oxidation kinetics differed from linear to parabolic rate in dependence on the fluid temperature [4]. They also presented a rate law in [4] to describe the oxidation kinetics with weight gain over exposure time. As described in reference [14], the weight gain of samples investigated in this work could not be used due to the limited dimensions of the cube specimen and therefore the potential high measurement error. For this reason, the following Equation (1) describes the increase in layer thickness instead of weight increase:

(1) $$L=k\cdot {\left(t\right)}^{n}$$

where L is the element layer thickness in nm; the rate constant k also has the SI unit nm. The variable t is exposure time in h divided by one hour so the dimensions remain correct after being raised by the power of n, which is also dimensionless in this work. Avelar et al. investigated AISI 304L and 348 in HTW and also found linear–parabolic rate kinetics [19]. Kuang et al. detected Cr oxides on AISI 304 when immersed into HTW, which gradually decreased while Fe oxides build up to a higher level [7]. In reference [16], it was found that a Ni content over 10 wt.% in AISI 316 lead to Ni oxides in the primary as well as the secondary oxide layer of the SS. Terachi et al. examined the morphology and formation process of surface oxides at different Cr contents [17]. They observed a dual-layer oxide structure and found that an increase in Cr content lead to a decrease in corrosion rate due to the Cr-rich primary layer acting as a diffusion barrier [17]. This dual-layer structure was also observed by reference [18]. Studies such as [4,16,20,21,22,23] investigated the protective Cr-rich primary oxide layer. Wang et al. found Cr-rich nano-crystalline structures in the primary layer of AISI 316 SS when he investigated the influence of surface finish on the corrosion behavior [20]. The influence of the surface finish of AISI 304L was investigated by reference [23]. Movahedi-Rad et al. investigated the oxidation behavior of austenitic AISI 321, 316 as well as ferritic 409 SS over exposure time in HTW [21]. They found that the austenitic SS had a lower oxidation rate compared to the ferritic SS [21]. However, no available work has been found on the primary oxide layer development over time in HTW of X6CrNiNb18-10.

This study investigates the exposure of X6CrNiNb18-10 to HTW for different durations. For this reason, this study focuses on the oxide morphology, growth, composition, and dimensions over exposure time, with particular attention to the time-dependent presence of Cr and Ni in the dual-layer structure. Therefore, scanning electron microscopy (SEM) and transmission electron microscopy (TEM) were used to investigate the samples exposed to HTW. In TEM, energy-dispersive X-ray spectroscopy (EDX) was used to measure element fractions of Cr, Fe, Ni, and Nb in correspondence to the change in the layer thickness of the dual layer. At the beginning of the experiment, the oxide phases’ determination was not the focus; however, since X-Ray Photoelectron Spectroscopy (XPS) measurements were conducted, the results are presented in this work. This experiment was only carried out once. No reproducibility analyses were carried out. The EDX line scans were distributed over the FIB cut lamella to record deviations in oxide layer growth.

## 2. Materials and Methods

### 2.1. Material and Specimen Manufacturing

The samples used in this work were taken from a X6CrNiNb18-10 pipe, also known as AISI 347 or 1.4550, which matches the standards for use in German NPP. X6CrNiNb18-10 is also known as AISI 347 or 1.4550. The chemical composition is given in Table 1. Manufacturing was conducted according to DIN 17458, with solution annealing at 1050 °C for a holding time of 10 min, followed by quenching in water. The microstructure and material properties of this material batch have already been presented in [24,25].

The samples were manufactured with a milling machine to approximate dimensions of 10 mm × 10 mm × 10 mm. Subsequently, the surface was polished to a roughness matching typical fatigue specimens using 1000 grit silicon carbide (SiC) papers. According to surface roughness measurements, this polishing procedure led to an average roughness value Ra of 0.200 µm for the sample exposed to HTW for 72 h, 0.202 µm for the sample exposed for 168 h and 0.180 µm for the sample exposed for 480 h.

### 2.2. High-Temperature Water Cycle

Before initiating the experiment, the samples were cleaned with alcohol and acetone. The samples were immersed in HTW for exposure times of 72 h, 168 h and 480 h. To replicate the influence of corrosion of SS in BWR NPP [26], the highly purified water was heated to 240 (±0.54) °C and pressurized to 7 (±0.07) MPa. This high temperature above the boiling point at an ambient pressure and pressure of 7 MPa allows the use of the expression HTW. The closed system had a volume flow of 16 L h^−1^. Dissolved oxygen was limited to 0.4 (±0.011) ppm by adding nitrogen to the water cycle. The conductivity was set to 0.055 µS cm^−1^, according to [11,27]. To guarantee the high purity of the HTW, it was continuously circulated in a purification circuit and cleaned via ion exchangers, mechanical microfilters, and activated carbon filters. Furthermore, the water was irradiated with UV light to prevent the formation of organic components. To ensure homogeneous flow conditions of HTW, a stack of SS (AISI 347) sample holders was constructed and placed in the 0.2 L autoclave. A Computational Fluid Dynamics (CFD) simulation predicted homogeneous flow conditions of 0.14 m s^−1^ [14]. After the samples had reached the predetermined exposure time, the autoclave temperature was reduced below to 100 °C within 60 min. Before sample extraction, the pressure was reduced to ambient pressure, the samples extracted, and the pressure increased. This process required 30 min, while the subsequent heating process required 120 min. After the required time in HTW, the samples were extracted, dried in air, and stored in a desiccator to avoid further oxidization.

### 2.3. SEM Observation

In order to observe the surface morphology of the oxidized samples, SEM analysis was conducted with a dual-beam system (Zeiss Auriga 40 FEG, Oberkochen, Germany) at an accelerating voltage of 7 kV. For subsequent investigations with TEM, the focused ion beam (FIB) of the Auriga system was used to cut into the surface. This procedure was explained in reference [20]. To protect the oxide film from sputtering, a Pt deposit was applied with dimensions of 2 µm × 2 µm × 25 µm on the area intended for the cut. Ga++ ions with a voltage of 30 kV and currents of 10 nA, 2 nA, 200 pA, and 50 pA were used to cut down the applied Pt deposit. This 2 µm Pt deposit was then thinned to 100 nm and cut free from the sample.

### 2.4. TEM Observation

Oxide layer determination was carried out in a JEOL-type JEM-2010F TEM (JEOL, Tokyo, Japan) with a field emission cathode (200 kV). The precipitates were identified by EDX mapping and spot measurements. For analytical studies, the TEM was equipped with an Apollo EDX system from EDAX. The detection limit was 0.1 wt.% and the spatial resolution was about 2 nm. The results were normalized to 100%, ignoring the light elements not detectable by this technique. The accuracy of EDX analysis was about 10% of the measured value. The accuracy of the EDX-analysis-measured distance was ±0.945 nm. However, the agreement between EDS measurements was much better, likely around 0.2% absolute. Element mapping was conducted for Fe, Cr, Nb, Ni, and O. Other elements were omitted, since the main focus of this work’s investigations was on the dimensions of secondary and primary oxide layers, defined by their Fe, Cr, and O fractions.

**Figure 1:**
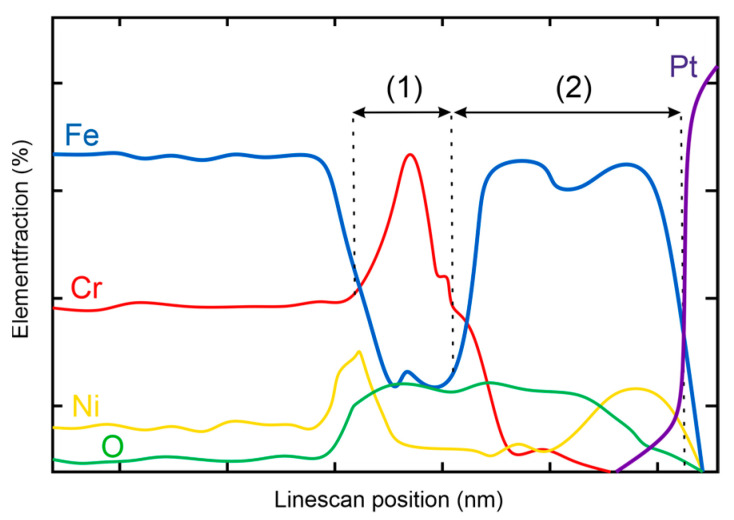
Schematic illustration of TEM EDX line scan results with two approaches to measure oxide layer thickness.

There are various ways to determine the oxide layer thickness. For example, Fulger et al. [4] considered the intersections of Fe with Cr. In contrast, Ling et al. considered the increase and subsequent development of oxygen in the elemental fraction [22]. Since the Ni present can also form a bond with the penetrating oxygen of the HTW, Cr can deplete into the secondary Fe-rich oxide layer as well as Ni. For this reason, the layer thickness of certain elements and corresponding development was measured in this work. The methodology of layer thickness determination is visualized in the schematic of TEM EDS line scans shown in Figure 1. Oxide compositions appearing first, such as Cr-oxide, were determined by measuring the inclination starting from the base line of non-oxidized metal to the point where the value of the preceding base line was reached again (1). Subsequently, secondary Fe-oxide layers show a low point in the element fraction due to element increase in the preceding primary oxide layer. The distance of this low point to the Pt layer was measured in this work (2).

### 2.5. XPS Analysis

To identify the oxide phases, XPS measurements were conducted with a Thermo VG Theta probe system (Thermo Fisher Scientific, Waltham, MA, USA). The monochromatic X-ray source produced Al Kα radiation (1486.68 eV) with an electrical power of 100 W. The X-ray beam size on the sample was 400 μm. The base pressure of the XPS analysis chamber was 3 × 10^−10^ mbar. For all measurements, a survey measurement was taken with a pass energy (PE) of 200 eV and five scans. The dwell time used was 50 ms, and the step size was 0.2 eV. For all single-scan measurements, the instrument’s snap scan mode with 500 scans was used, each lasting 1 s. Data analysis was performed with the Powell-fitting algorithm and smart-background subtraction, integrated into the XPS software “Avantage” (www.thermofisher.com, accessed on 1 September 2024). The calibration of the measured binding energy was performed and periodically checked with a silver calibration sample. The measured peak position was compared with the nominal one.

## 3. Results

**Figure 2:**
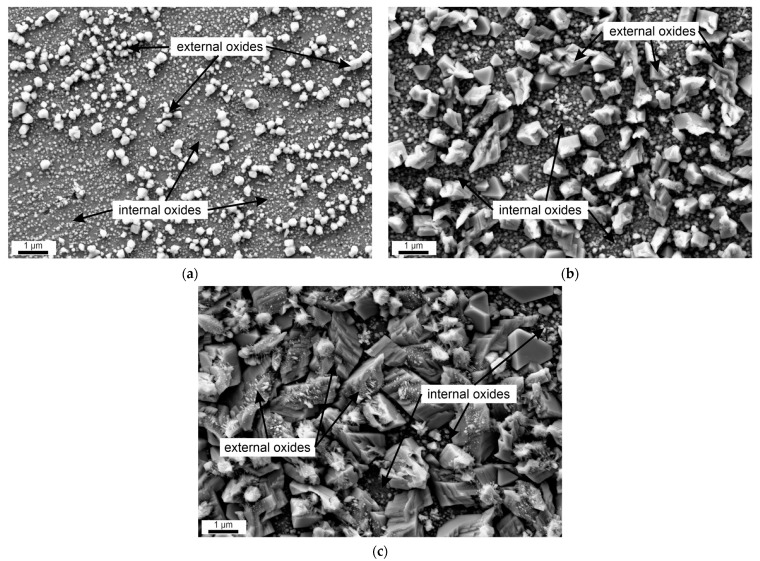
SEM images of the oxidized surface after (**a**) 72 h, (**b**) 168 h and (**c**) 480 h exposure to HTW.

The surface morphologies of the oxide film observed using SEM are illustrated in Figure 2a after 72 h of exposure, after 168 h in Figure 2b and after 480 h in Figure 2c, respectively. As shown in Figure 2, after 72 h, the surface is sparsely covered with oxides. The oxides partially show a polyhedral form. By the end of 168 h in HTW, the typical dual-layer structure of the formed oxides can be observed (see Figure 2b). Here, two characteristic crystallin forms, grown slightly greater than 1 µm, are visible in the secondary outer layer, while the lower layer is characterized by nanocrystals. After 480 h, the secondary layer shown in Figure 2c almost completely covers the surface and only small regions of the nanocrystalline primary layer are visible. Comparing the diameter of the nanocrystals in the primary layer after 168 h (Figure 2b) and after 480 h (Figure 2c), no significant increase can be observed. However, the secondary layer exhibits significantly larger oxides. While the polyhedral crystals only grew in size, other crystals showed a more irregular shape with a more gaped surface seeming to have formed a bond with other oxide compounds.

The chemical composition of the oxides on the surface can be analyzed in depth with the FIB cuts examined in TEM, and the thereby-obtained element maps of Fe, Cr, Nb, Ni and O. Although Nb is present only in small amounts in the material, EDX line scans and element maps are also examined for it, as this distinguishes the investigated material from other austenitic stainless steels such as AISI 304 or AISI 316. Furthermore, the corresponding EDX line scans provide additional data for the analysis of the layer and oxide composition. For this reason, the EDX line scans start from the matrix and go to the applied Pt layer.

**Figure 3:**
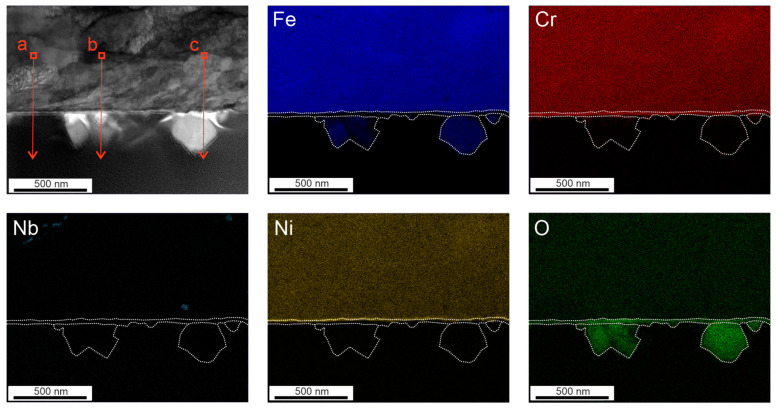
Oxide film formed after 72 h in HTW with corresponding element maps of Fe, Cr, Nb, Ni and O, including the path of EDX line scans illustrated as a red arrow, performed by TEM.

**Figure 4:**
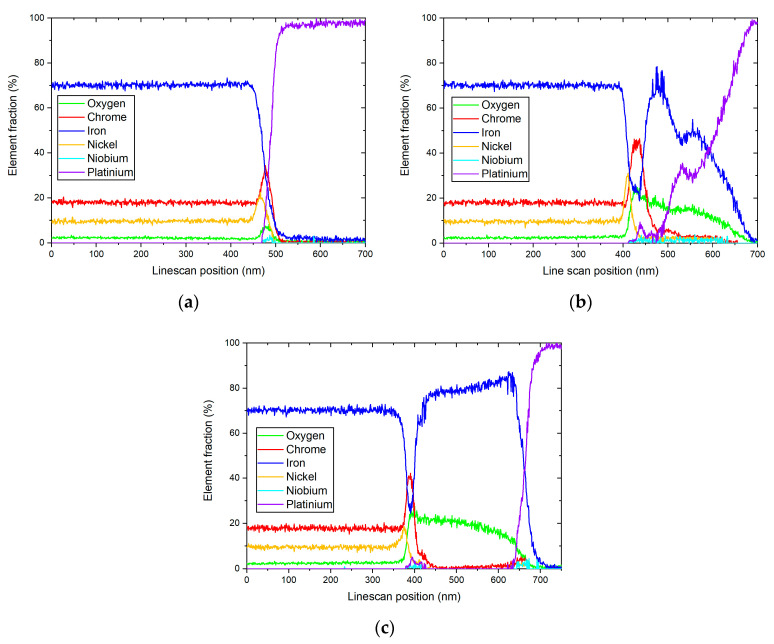
Results of corresponding EDX line scans of a specimen after being exposed to HTW for 72 h with reference to the markings in Figure 3.

Figure 3 shows the element maps after 72 h of exposure in HTW. The secondary oxides consist of Fe. Ni was segregated to the interface between the Cr oxide layer and the matrix. A difference between the secondary oxides can be seen. The primary Cr-rich oxide layer presents itself as uniform. The three EDX line scans were carried out on the depicted section in Figure 3 and are illustrated in Figure 4. While the Cr oxide layer shows a predominantly constant thickness of 30 to 48 nm, the Ni layer is less enriched under the crystal on the right side, with 17.59% (see Figure 4c) compared to the 22.4 and 31.1% in Figure 4a,b. An impact on the thickness of the Ni-enriched layer could not be observed in Figure 4. However, the high Ni peak of 31.1% (see Figure 4b) was followed by a Ni peak of 2.983% in the secondary layer. Line scans with lower element fraction (Figure 4a,c) detected no Ni peak in the secondary layer. The element map of Figure 3 shows an increase in oxygen where a secondary oxide layer is present. The diffusion of Cr and Ni to the secondary oxide layer cannot be anticipated from the color maps in Figure 3. However, the line scans show the presence of Cr, even if only to a minor fraction of 5.25%, in Figure 4c.

**Figure 5:**
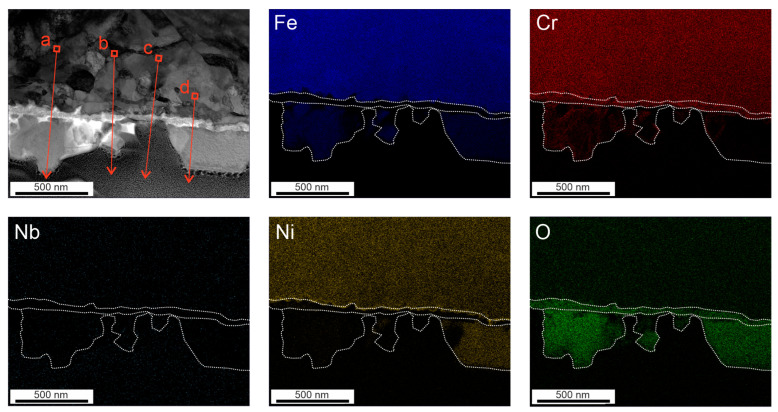
Oxide film formed after 168 h in HTW with corresponding element maps of Fe, Cr, Nb, Ni and O, including the path of EDX line scans illustrated as red arrows (performed by TEM).

**Figure 6:**
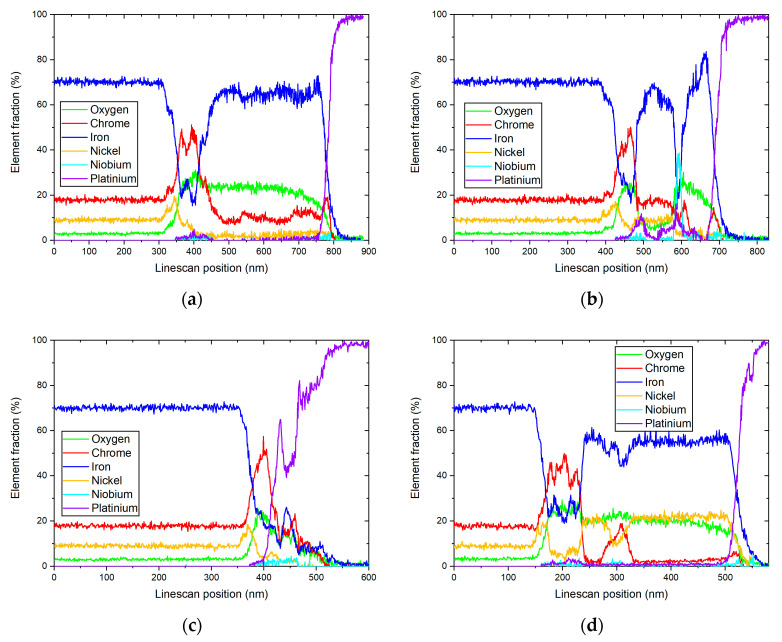
Results of corresponding EDX line scans of a specimen after being exposed to HTW for 168 h with reference to the markings in Figure 5.

After 168 h in HTW, the presence of Cr in the secondary oxide layer can be observed in Figure 5. The increased presence of oxygen under secondary oxides, as observed after 78 h, is also present in the element maps of Figure 5. Four EDX line scans were carried out, all of which detected Cr in the secondary oxide layer. It is noticeable that the Fe oxides on the left side are associated with Cr, while the oxide crystal on the right side is rich in Ni. In Figure 6d, the Ni element fraction is up to 24.81%, which is higher than in the matrix with 10%. A difference in layer thickness regarding Ni can also be observed. Figure 6a illustrates a Ni layer thickness of 53 nm, and Figure 6b one of 55 nm. In contrast, the layer thickness of the Ni-enriched measurements in Figure 6c show 31 nm and in Figure 6d show 33 nm. Nevertheless, no correlation of increased Ni element fraction and the presence of Ni in the secondary layer can be observed in the specimen after 168 h in HTW. Ni peaks above 20% are not present before the Cr-rich layer, but can be seen in some oxides of the secondary oxide layer (see Figure 6d). In addition, Figure 6b indicates an increase in Nb element fraction up to 38.6% which, at this high value, is not related to the usual deflections associated with measurements near the Pt layer of Nb up to 4%. As oxidation progresses, the influence of grain boundaries can also be seen in the more irregular development of Cr depletion and Ni enrichment at the primary layer, illustrated in Figure 5. This impacts the thickness of the primary protective oxide layer by being rather non-uniform.

**Figure 7:**
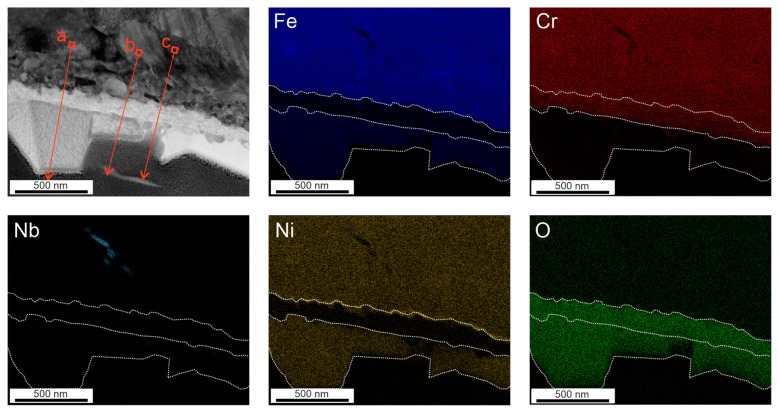
Oxide film formed after 480 h in HTW with corresponding element maps of Fe, Cr, Nb, Ni and O, including the path of EDX line scans illustrated with red arrows (performed by TEM).

**Figure 8:**
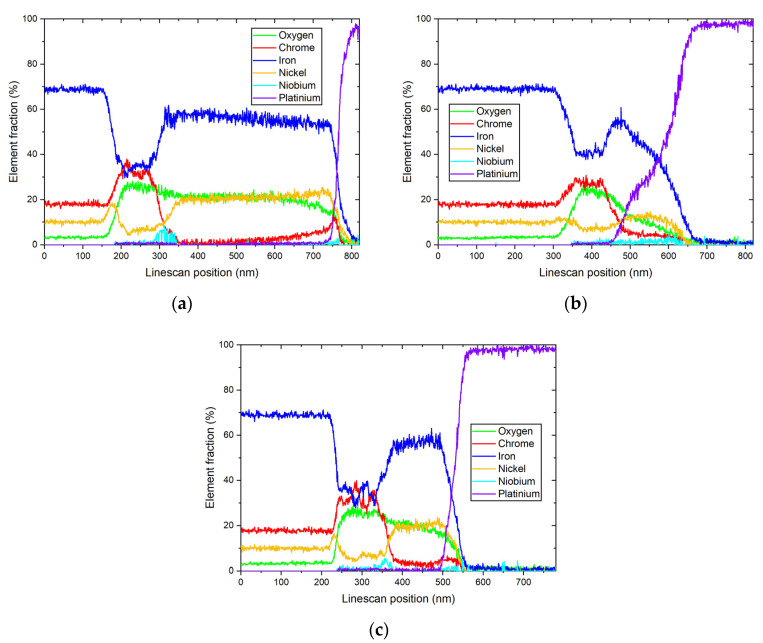
Results of corresponding EDX line scans of a specimen after being exposed to HTW for 480 h with reference to the markings in Figure 7.

The irregular development of Cr depletion and Ni enrichment is also present in the observed FIB cut after 480 h, as shown in Figure 7. Furthermore, all oxides in the secondary layer show an Fe–Ni bond visible in Figure 7. Only the EDX line scans of Figure 8 show that Cr is still partially present in the structure. Especially below and on the Ni–Fe oxide, the Cr content in Figure 8a,c increases with smalls peaks of 10.38% and 5.88%. However, the element fraction of Cr in the secondary Fe-rich layer is lower compared to 168 h. Additionally, the element fractions of Cr in Figure 8 are all <40%, while after 168 in Figure 6 the measured Cr was >40%. Similar to the specimen after 168 h, the Ni-enriched zone under secondary oxides increased by 39 nm (see Figure 8a) and 40 nm (see Figure 8b), compared to the 29 nm observed in Figure 8c. With the exception of the line scan of Figure 8b, Ni content in the secondary oxide layer was higher than in the matrix (see Figure 8a,c). It is noteworthy that Nb peaks were again detected in the secondary oxide layer shown in Figure 8a,c, without an increase in Pt measurement. In addition, in the element map of Figure 3 and Figure 7, where NbC can be observed in the metal matrix, Nb is only detected via EDX line scan.

**Figure 9:**
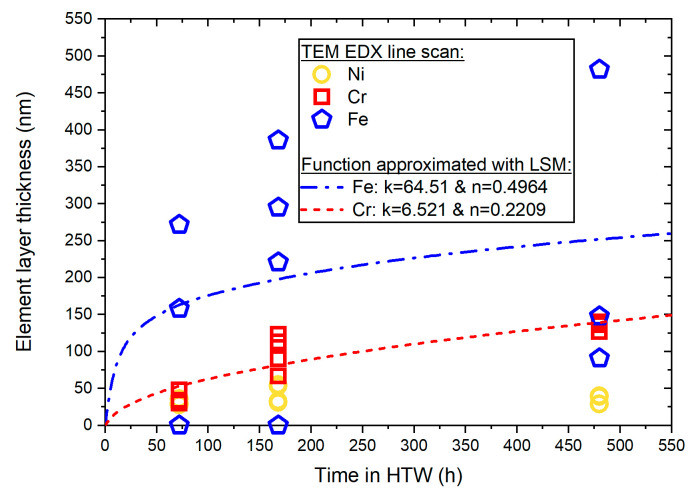
Element layer thickness of Cr, Ni and Fe over time in HTW with parabolic functions to replicate the oxide layer growth over exposure time, approximated with the least squares method.

To analyze the development of Cr, Ni and Fe layer thickness, the element fractions of the EDX line scans (Figure 4, Figure 6 and Figure 8) were evaluated according to the methodology described in Figure 1. The resulting values over time spent in HTW are illustrated in Figure 9.

**Figure 10:**
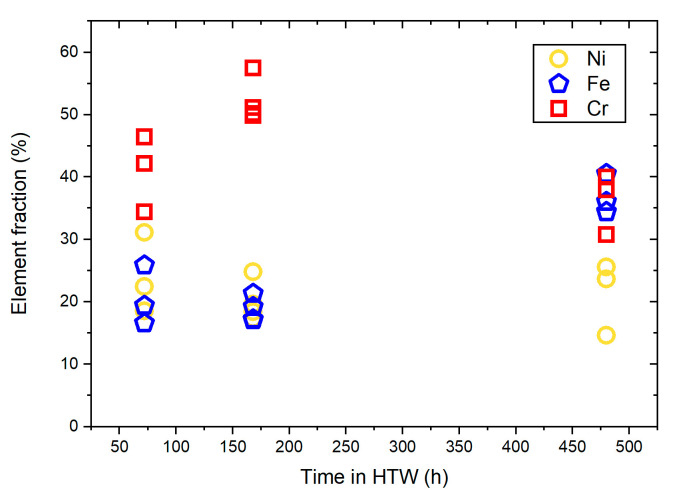
Maximum fraction of elements in inner layer measured with EDX line scans.

The latter were used to determine mean values with their standard deviation. Cr showed a parabolic development. After 78 h in HTW, the protective Cr layer’s average thickness was 37.33 ± 7.71 nm, and 98.25 ± 21.65 nm after 168 h and 134.3 ± 5.43 nm after 480 h. After 78 h in HTW, the average Fe layer thickness was 143.3 ± 111.5 nm. This Fe layer increased to 225.5 ± 142.7 nm after 168 h in HTW, and 240.3 ± 172.5 nm after 480 h. Regarding Ni, a more constant development was present, with 31.33 ± 3.4 nm after 78 h and 43 ± 11.1 nm after 168 h, declining to 36 ± 4.97 nm after 480 h. In Figure 10, the maximum element fraction in the inner layer of Figure 4, Figure 6 and Figure 8 over exposure time in HTW is summarized.

**Figure 11:**
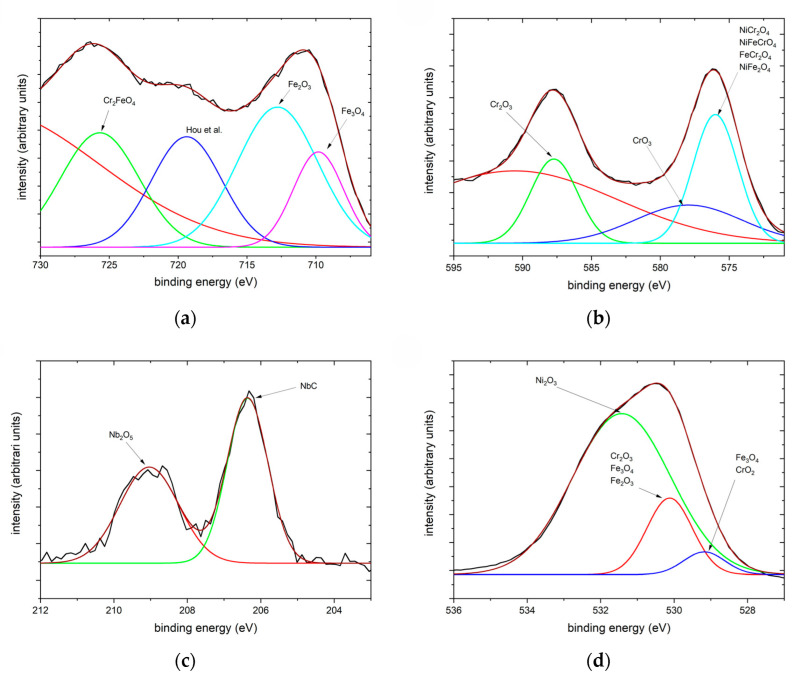
XPS spectra of (**a**) Fe2p3/2, (**b**) Cr2p3/2, (**c**) Nb3d5/2 and (**d**) O1s after 20 days in HTW with the measured signal as black line and the accumulated approximation peaks as red line [29].

Figure 11 shows the XPS spectra of the oxide film formed on the surface of the X6CrNiNb18-10 specimen after 20 days in HTW. Although the identification of the oxide phases is not the main focus of this work, the NIST X-ray Photoelectron Spectroscopy Database [28] was used to evaluate the peaks. Since this identification is based on a comparison of the peaks to literature values, an evaluation of the results is conducted in the Discussion section.

## 4. Discussion

No available literature could be found regarding the primary oxide layer development of AISI 347 in BWR. In this aspect, the results of this work are a novelty. The present oxide layer development with its dual-layer structure over exposure time in HTW is comparable to the references of [4,7,16,17,18] regarding other SS with similar chemical composition, such as AISI 304 or AISI 316. The morphological development of oxides observed with SEM (in Figure 2) is basically consistent with the described oxidation process of [7], with initial hematite nucleation followed by growth and resulting in a compact secondary layer. The presence of a dual layer is based on the difference in electrochemical potential due to changes in oxygen content in the oxide layer [17]. Different growth rates presented in the literature are attributable to different medium flow rates. While the authors of [4,22] used a static tank, flow conditions varied in other references [7,16,18,23,30]. The flow conditions in this work were also attributed to the increased progression of corrosion compared to quasi-static flow conditions, as can be seen in [14]. Furthermore, differences in the HTW chemistry to align conditions of PWR [16,23,30,31], BWR [18,23,32,33,34] and others [4,35] influence the oxides’ morphology and growth, especially dissolved oxygen [31,36]. In [20,23,37], the influence of surface roughness on the oxide layer development is described. However, it was ensured that the specimen in this work showed an almost identical surface finish, and thus this influence can be neglected. Another influence on the known deceleration [4] of oxidation in SS is the secondary oxide layer itself. The secondary oxide layer is also known to serve as some kind of protective layer [13]. The results depict the progress of secondary oxide layer growth which thereby increases the surface roughness of the former smooth sample surface. Oxides growing from nano- to microscale lead to an increased surface roughness of samples in HTW. According to [38], increased surface roughness leads to an increased boundary layer. The present flow conditions are thus converging locally towards the flow conditions of quasi-static tanks. Examining the maximum height of the oxides in Figure 4c with 268 nm, Figure 6a with 375 nm and Figure 8a with 482 nm, this growth is perceptible [12]. This can also be seen in Figure 9, with the corresponding layer thickness of Fe. When comparing with the literature, the different chemical compositions of SS [16,36], as well as local inhomogeneities or different phases like austenite and δ-ferrite [30], must be considered. Regarding this work, an identical batch of X6CrNiNb18-10 was exposed to HTW and no influence of δ-ferrite could be observed.

Since Ni remained nearly constant over exposure time, it was not approximated with Equation (1). Fe and Cr were approximated using the least squares method (LSM) and the measured element layer thickness over exposure time in HTW (see Figure 9). The results of the approximation were added to Figure 9. For Fe, the rate constant k was 64.51 nm, while n was 0.2209. According to reference [4], this indicates cubic oxide growth of the Fe layer. For Cr, the rate constant k was 6.521 nm and n equaled 0.4964, which indicates parabolic oxidation kinetics [4]. Parabolic oxide layer development, illustrated in Figure 9, is in accordance with references [4,21,32,39]. Additionally, the chemical composition in the initial state (Table 1) matches the magnitude measured with the EDX line scan (see Figure 4, Figure 6 and Figure 8). The results shown in Figure 3, Figure 5 and Figure 7 revealed the composition of secondary oxides on the samples surface. Both the element maps and the EDX line scans of Figure 6 and Figure 8 show the increasing Ni content in the secondary Fe-rich oxide layers over exposure time in HTW. Comparable Ni accumulation in the secondary Fe-rich oxide layer was not observed by Lin et al. [30] for AISI 308L exposed to PWR conditions or Fulger et al. [4] for 304 SS in SCW. However, refs. [16,40] presented Ni-enriched secondary oxides in their observations of 304 and 316 SS in PWR, with an element fraction of Ni close to the sample after 168 h in this work. Warmbach et al. also published the results of type 316 SS after 30 days in BWR HTW medium conditions with Ni in the secondary oxide layer. However, the magnitude of volume flux with 1 L h^−1^ [41] was comparably small and thus oxidation was not as progressed as in the 480 h sample of this work. Kuang et al. [31] investigated AISI 304 in HTW and found a correlation of Ni with dissolved oxygen and exposure time, which explains previously mentioned differences in the literature. As also described by [31], the binding of Ni as an oxide requires the presence of hematite. This explains the development of Ni in the secondary oxide layer with the increasing volume of the Fe-rich secondary layer over exposure time in HTW. Ni in the secondary layer has also been presented by Wang et al. [20] for 316 SS after 120 h in PWR conditions with similar element fractions. In reference [23], it is pointed out that secondary oxides in a PWR environment often show no Ni fraction. An increasing Ni element fraction over exposure time can be observed in the secondary oxide layer in Tapping et al. [39]’s study for 304 SS in PWR HTW, correlating with a reduction in Cr. Wang et al. [20] also showed Cr in the secondary oxide layer. In reference [18], the depletion of Cr in the outer secondary layer in BWR conditions is reasoned with the further oxidation of chromium. This would explain the reduction in the Cr element fraction in this work (see Figure 6 and Figure 8) from 168 h to 480 h in the outer oxide layer. However, the exact element fraction is not given in reference [20] for comparison with the results of this work. Terachi et al. [17] investigated the influence of chromium content on the oxide layer developed in 316 SS. With increasing chromium content, not only was the thickness of oxide layers influenced, but also the structure of the secondary oxide film, even resulting in FeCr_2_O_4_ and NiFe_2_O_4_ as well as NiCr_2_O_4_ [17].

Considering the XPS results in Figure 11b, with a peak at 576 eV, the presence of NiCr_2_O4, NiFeCrO_4_ and FeCr_2_O_4_ can be confirmed with Allen et al. [42] as well as NiFe_2_O_4_ with reference [43]. Whilst the peak at 530.12 eV can suggest Cr_2_O_3_ [44] or Fe_3_O_4_/Fe_2_O_3_ [45,46] and the peak at 529.18 eV Fe_3_O_4_ [47] or CrO_2_ [48], the peak at 531.42 eV implies Ni_2_O_3_ [49]. The presence of Fe_2_O_3_ or Fe_3_O_4_ can be analyzed with the peaks in Figure 11a at 712.82 eV [45] and 709.82 eV [44,50]. A peak at 719.38 eV can be reasoned with the findings of Hou et al. [29]. In addition to the already-mentioned elemental composition with Cr, the peak at 725.69 eV of Figure 11a suggests Cr_2_FeO_4_, as in accordance with Langevoort et al. [51]. Furthermore, peaks at 587.73 eV and 577.97 eV of Figure 11b indicate Cr_2_O_3_ [52] as well as CrO_3_ [53]. The results of the XPS analysis are also in accordance with references [4,7]. Selected area diffraction could give a more precise statement of the oxide phases in the specific oxides. However, the main focus of this work lies in the determination of oxide layer dimension development over HTW exposure time.

The occurrence of Nb in the secondary oxide layer is striking (see Figure 6b and Figure 8a). Typical austenitic stainless steels used in NPP contain no Nb (see references [4,20,54]), or at least not to a comparable amount (see reference [30]), as it is included in this material with 0.62 wt.%. For this reason, Nb was not investigated to a comparable detail as Ni or Cr. Nb is present in the investigated material of this work in the form of NbC (see Figure 3 and Figure 7), as well as in the peak at 206.36 eV of Figure 11c, which is in accordance with Silva et al. [55]. It cannot be ruled out that parts of NbC, which was originally present in the matrix and thus may also be present on the sample surface, were removed by the oxidation process due to the associated Cr and Ni enrichment. According to EDX line scans in this work, Nb element fraction often increases before secondary oxides are present. It cannot be ruled out that NbC could also serve as the nuclei for the oxides of the secondary layer. Since Nb does not seem to have a major influence on the oxidation layer development studied here, it is not investigated further and reference is made to the work of Blackburn et al. [56] dealing with the reaction of Nb and oxygen in water vapor. However, considering the XPS results from Figure 11c, it can be stated that the Nb reacted with oxygen to form Nb_2_O_5_, due to the presence of a peak at 209.04 eV, as in references [57,58]. To the authors’ best knowledge, this is the first time niobium pentoxide has been detected in a BWR HTW environment. Considering the fact that niobium serves to stabilize the material, this finding is particularly significant. If one considers the EDX line scans after 72 h in Figure 4 to the 480 h exposure time in Figure 8, it can be found that the quantity of occurrence and magnitude in the element fraction increases over exposure time, regarding the secondary oxide layer. Whilst the color maps of Figure 3 and Figure 5 do not indicate a significant Nb accumulation in the outer oxide layer, Figure 7 displays Nb accumulation directly after the primary oxide layer and at the edge of Ni- and Fe-rich oxides, not within them.

Regarding the primary oxide layer of this work (see Figure 3, Figure 4, Figure 5, Figure 6 and Figure 7), reference [17] does not show a Cr-depleted zone. The EDX line scans of Figure 4, Figure 6 and Figure 8 do not show Cr-depletion, but a reduction in Fe in the area of Ni-enrichment. This is in accordance with Xiao et al. [16], who investigated 316 SS, and Tapping et al. [39] for 304 SS. For this reason, it is more appropriate to speak of an enrichment rather than a depletion zone with respect to Ni and Cr. Regarding 304 SS in BWR and PWR conditions, reference [23] shows similar color maps regarding Ni and Cr. However, the layer thickness was analyzed using XPS sputtering and is therefore given in sputtering time and not nm. For this reason, no comparison to the element fraction of Jaffeé et al. [23] was conducted. Nevertheless, Jaffeé et al. [23] measured a total equivalent oxide layer thickness after 120 h in medium conditions (BWR) at a flow rate of 10 L h^−1^ with 120–218 nm depending on the surface finish. If one compares these values to the data of Figure 9, and neglects possible difference in local flow conditions, AISI 347 and 304 SS reach similar states. Degueldre et al. also investigated the growth of the oxide layer with an in-line diffuse reflection spectroscopy study and state quite similar to the development of oxide layer thickness as a function over time for 316 SS in BWR [33,34]. This further confirms the validity of the experiments presented in this work. In addition, reference [39] also showed no depletion of Cr with increasing Ni fraction, but a reduction in Fe concentration. Furthermore, regarding the element fractions of Cr, Fe and Ni in the inner layer over time, the results of the EDX line scans are summarized in Figure 10 for comparison to the XPS results of type 304 SS of Tapping et al. [39]. While the development of Cr element fraction can be compared to the observations of Tapping et al. [39], the magnitude of the measured values, as well as Fe and Ni development, differ due to the PWR medium conditions, local flow conditions and difference in chemical composition of the SS. In addition, Tapping et al. only saw increasing oxide layer growth due to the primary layer and not due to the secondary Fe-rich layer, which underlines the differences and deviations described previously [39]. The element fractions of Figure 10 are more comparable to the magnitudes published by Stellwag [12], who names Cr_2_O_3_, Fe_2_CrO_4_, FeCr_2_O_4_ and NiCr_2_O_4_ as bonds for the chromium-rich spinel of the inner layer. The aforementioned bonds are also in accordance with references [18,27,41] regarding SS in BWR and, as described previously, can be observed in the XPS results of Figure 11 of this work. Moreover, the thickness of the inner layer after 72 h with 30 to 48 nm and 67 to 123 nm after 178 h (see Figure 9) is also in accordance with the 50 to 100 nm published by Stellwag [12] for SS. After 480 h in HTW conditions the growth of the Cr-rich protective inner layer thickness amounts to significantly more with 127 to 140 nm, as growth has continued despite the saturation of development, as can be expected for parabolic growth. 

Overall, X6CrNiNb18-10 showed a similar oxidation behavior compared to other type-300 SS. The high local velocity of the HTW accelerated the rate of corrosion development compared to the results of other published works for static tanks or other velocities. Furthermore, for an exact comparison, identical conditions of pressure, temperature and dissolved oxygen are required. For this reason, further experiments with nuclear grade AISI 304 and ER 347 (weld metal) are intended. Furthermore, fatigue tests with an AISI 347 specimen pre-exposed to HTW over 420 h are of interest, due to the possible influence of the niobium pentoxide formation on the Nb-stabilized SS and its impact on material degradation, as well as on possible intergranular stress corrosion cracking [2,7,8,9,11]. The determined oxidation kinetics of SS primary and secondary oxide layer can be used for the numerical modeling of corrosion cracking, as in Li et al. [59].

## 5. Conclusions

In this work, the oxide layer development of X6CrNiNb18-10 over exposure time in HTW has been investigated. The development of the protective primary oxide layer dimensions has been successfully characterized. The following conclusions can be drawn from the presented results:

- A parabolic development of Cr in the protective primary layer and a cubic oxidation rate of Fe in the secondary layer were observed, but the dimensions of the Ni layer remained constant.
- After 78 h in HTW, the protective Cr layer’s average thickness was 37.33 nm, and 98.25 nm after 168 h and 134.3 nm after 480 h. The average Fe layer after 78 h was 143.3 nm, and 225.5 nm after 168 and 240.3 nm after 480 h in HTW. Regarding Ni, a more constant development was present, with 31.33 nm after 78 h, 43 nm after 168 h, and 36 nm after 480 h in HTW.
- Ni required the presence of a pronounced Fe-rich secondary layer before being able to reside on the outer secondary layer. With increasing immersion time, the Ni element fraction surpassed the Cr element fraction in the secondary layer. After 480 h, almost the entire surface was covered by the outer oxide layer.
- In the metal matrix, no depletion of Cr or Ni could be observed over time. However, an increased presence of Cr and Ni in the primary layer was observed, to the expense of Fe content.
- Some Nb, added to the alloy for stabilization, reacted to niobium pentoxide (Nb_2_O_5_) on the specimen surface after exposure in HTW. The quantity and magnitude increased over exposure time. With regard to the long-term operation extension of BWR NPP, this finding is of special significance due to the possible impact on environmental fatigue behavior of the SS.

## Figures and Tables

**Table 1:** Chemical composition of X6CrNiNb18-10 stainless steel (wt.%).

C	Si	Mn	Mo	P	S	Cr	Cu	Ni	Nb	N
0.040	0.41	1.83	0.29	0.02	0.007	17.6	0.06	10.6	0.62	0.007

## Data Availability

Raw or processed data maybe shared upon request to the corresponding author after approval of the MPA directors, the project executing agency (GRS) and the German Federal Ministry for Environment, Nature Conservation, Nuclear Safety and Consumer Protection.
